# Data on assessment excess lifetime cancer risk and risk of lung cancer from inhalation of Radon 222 in radiotherapy centers in Tehran, Iran

**DOI:** 10.1016/j.dib.2018.09.005

**Published:** 2018-09-06

**Authors:** Mohammad Mirdoraghi, Safdar Masoumi, Daniel Einor

**Affiliations:** a Department of Radiology and Radiotherapy, School of Allied Medicine, Tehran University of Medical Sciences, Tehran, Iran; b Department of Epidemiology and Biostatistics, School of Public Health, Tehran University of Medical Sciences, Tehran, Iran; C Department of Biological Sciences, University of South Carolina, United States

**Keywords:** Background radiations, Excess lifetime cancer risk, Risk of lung cancer, Radiotherapy centers

## Abstract

The purpose of the data was to determine excess lifetime cancer risk (ELCR) and risk of lung cancer from inhalation of radon in radiotherapy staff at Tehran radiotherapy Centers in 2015.The concentration of radon gas was extracted from a study done at Tehran radiotherapy centers, and then ELCR and risk of lung cancer were calculated in all centers by standard equations. The excess lifetime cancer risk and risk of lung cancer were 1.89 and 8.46 cases per 100,000 people in radiotherapy centers in Tehran City. The data indicate that the excess lifetime cancer risk and risk of lung cancer in radiotherapy centers are lower than the standard values which presented by UNSCEAR 2000.

**Specifications table**

**Table:** 

Subject area	Radiation biology and radiation protection.
More specific subject area	Excess lifetime cancer risk and risk of lung cancer from inhalation of radon-222
Type of data	Tables, graph.
How data was acquired	The concentration of radon gas was extracted from a study done at Tehran radiotherapy centers [3], then the excess lifetime cancer risk and risk of lung cancer were calculated in all centers using standard equations [5], [6]
Data format	Analyzed.
Experimental factors	The concentrations of radon gas were analyzed according to the standards to calculate excess lifetime cancer risk and risk of lung cancer from inhalation of radon-222.
Experimental features	Excess lifetime cancer risk and risk of lung cancer from inhalation of radon-222 were determined.
Data source location	Tehran city, Iran.
Data accessibility	The data are available with this article

**Value of the data**

• Data showed that the excess lifetime cancer risk and risk of lung cancer in radiotherapy centers are lower than the standard values which presented by UNSCEAR 2000. That means the possible hazards from radon concentration are low compared to UNSCEAR 2000.
• Data can be used to demonstrate that the risk of lung cancer is greater than excess lifetime cancer risk in radiotherapy centers in Tehran City i.e., for the current population radon concentration should also be considered a potentially significant cause of lung cancer which is exposed through contamination of indoor air by radon from surrounding materials.
• The data can be used to compare ELCR and the risk of lung cancer with other studies in radiotherapy centers.

## 1 Data

**Diagram 1:**
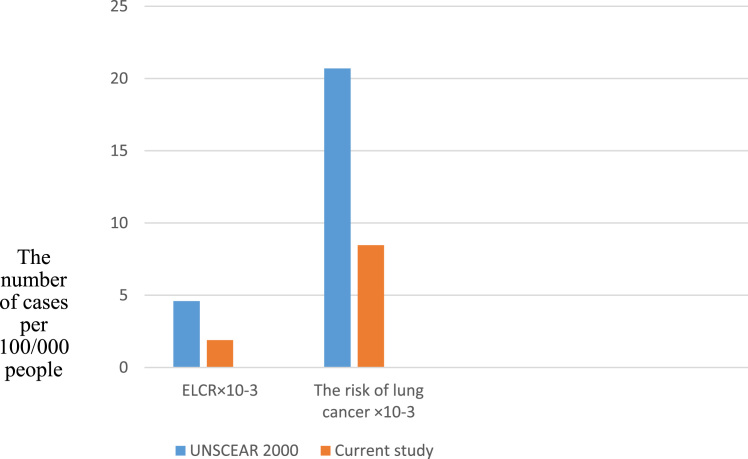
The comparison between ELCR and risk of lung cancer (×10^−^^3^) in the current study with 95% confidence intervals (CI) and the UNSCEAR 2000 value.

The excess lifetime cancer risk and risk of lung cancer were calculated in eight radiotherapy centers in Tehran (Table 1). The ELCR and the risk of lung cancer were compared with UNSCEAR 2000 range (Diagram 1). According to the UNSCEAR 2000 the annual effective dose for radiation workers by Radon-222 and Radon-220 is different from to 0.1 to 1.15 mSv [1], [2]. In this data, the mean annual effective dose is equal to 0.48 mSv.

**Table 1:** The excess lifetime cancer risk (ELCR) and risk of lung cancer (×10^−3^).

**Radiotherapy centers**	**Mean effective dose (mSv)**	**Standard deviation**	**ELCR×10^−3^**	**The risk of lung cancer per 100/000 people (×10^−3^)**
1	0.09	0.07	0.359	0.162
2	0.21	0.06	0.839	0.378
3	0.23	0.16	0.919	0.414
4	0.43	0.06	1.718	0.774
5	0.23	0.21	0.919	0.414
6	1.28	0.83	5.114	2.304
7	1.04	0.49	4.155	1.872
8	0.28	0.08	1.118	0.504
Total	3.79	0.35	15.08	6.79

## 2 Experimental design, materials, and methods

The concentration of Radon-222 was extracted from a study, which was carried out at eight radiotherapy centers in Tehran, Iran [3]. Then, the excess lifetime cancer risk and risk of lung cancer were calculated.

### 2.1 Assessing the excess lifetime cancer risk

To calculate the excess lifetime cancer risk due to gamma-ray radiation the following equation was used [4], [5], [6]:

(1) ELCR=AED×DL×RF

ELCR = Excess Lifetime Cancer Risk per 100/000 people, E = Annual effective dose in msv, DL = Average lifespan (year) = 70/1 years [7]. RF = fatal cancer risk per Sievert, risk factor (Sv^−1^) = 0/057 Sv^−1^. [8]

### 2.2 Calculating the risk of lung cancer

The probability of annual lung cancer cases per million people (CPPP) caused by effective dose received from Radon-222 was assessed by Eq. (2) [9], [10], [11]

(2) CPPP=ERn×18

ERn = Effective dose received by the Radon 222.
