# Supplementary material for: Data on assessment excess lifetime cancer risk and risk of lung cancer from inhalation of Radon 222 in radiotherapy centers in Tehran, Iran
Source: Data Brief. 2018 Sep 6;20:1367–70. doi: 10.1016/j.dib.2018.09.005 (PMC6148725; doi:10.1016/j.dib.2018.09.005)
Supplement: Supplementary file 1 — Supplementary material [file mmc1.docx]

To the editor, 26^th^ August 2018

**Data in Brief**

Dear editor,

There is no any conflict of interest for the manuscript entitled, “**Data on Assessment Excess Lifetime Cancer Risk and Risk of Lung Cancer from Inhalation of Radon 222 in radiotherapy centers in Tehran, Iran*”*** All authors declare that.

Thank you in advance for receiving our manuscript to review and your valuable time. Looking forward to hear from you soon.

Sincerely Yours,
*Mohammad Mirdoraghi*
